# Upper-Room Ultraviolet Light and Negative Air Ionization to Prevent Tuberculosis Transmission

**DOI:** 10.1371/journal.pmed.1000043

**Published:** 2009-03-17

**Authors:** A. Roderick Escombe, David A. J Moore, Robert H Gilman, Marcos Navincopa, Eduardo Ticona, Bailey Mitchell, Catherine Noakes, Carlos Martínez, Patricia Sheen, Rocio Ramirez, Willi Quino, Armando Gonzalez, Jon S Friedland, Carlton A Evans

**Affiliations:** 1 Department of Infectious Diseases & Immunity, Imperial College London, United Kingdom; 2 Wellcome Centre for Clinical Tropical Medicine, Imperial College London, United Kingdom; 3 Asociación Benéfica PRISMA, Lima, Perú; 4 Universidad Peruana Cayetano Heredia, Lima, Perú; 5 Johns Hopkins Bloomberg School of Public Health, Baltimore, Maryland, United States of America; 6 Hospital Nacional Dos de Mayo, Lima, Perú; 7 Universidad Nacional Mayor San Marcos, Lima, Perú; 8 Agricultural Research Service, U.S. Department of Agriculture, Washington, D. C., United States of America; 9 School of Civil Engineering, University of Leeds, Leeds, United Kingdom; University College London, United Kingdom

## Abstract

**Background:**

Institutional tuberculosis (TB) transmission is an important public health problem highlighted by the HIV/AIDS pandemic and the emergence of multidrug- and extensively drug-resistant TB. Effective TB infection control measures are urgently needed. We evaluated the efficacy of upper-room ultraviolet (UV) lights and negative air ionization for preventing airborne TB transmission using a guinea pig air-sampling model to measure the TB infectiousness of ward air.

**Methods and Findings:**

For 535 consecutive days, exhaust air from an HIV-TB ward in Lima, Perú, was passed through three guinea pig air-sampling enclosures each housing approximately 150 guinea pigs, using a 2-d cycle. On UV-off days, ward air passed in parallel through a control animal enclosure and a similar enclosure containing negative ionizers. On UV-on days, UV lights and mixing fans were turned on in the ward, and a third animal enclosure alone received ward air. TB infection in guinea pigs was defined by monthly tuberculin skin tests. All guinea pigs underwent autopsy to test for TB disease, defined by characteristic autopsy changes or by the culture of Mycobacterium tuberculosis from organs. 35% (106/304) of guinea pigs in the control group developed TB infection, and this was reduced to 14% (43/303) by ionizers, and to 9.5% (29/307) by UV lights (both *p* < 0.0001 compared with the control group). TB disease was confirmed in 8.6% (26/304) of control group animals, and this was reduced to 4.3% (13/303) by ionizers, and to 3.6% (11/307) by UV lights (both *p <* 0.03 compared with the control group). Time-to-event analysis demonstrated that TB infection was prevented by ionizers (log-rank 27; *p <* 0.0001) and by UV lights (log-rank 46; *p <* 0.0001). Time-to-event analysis also demonstrated that TB disease was prevented by ionizers (log-rank 3.7; *p =* 0.055) and by UV lights (log-rank 5.4; *p =* 0.02). An alternative analysis using an airborne infection model demonstrated that ionizers prevented 60% of TB infection and 51% of TB disease, and that UV lights prevented 70% of TB infection and 54% of TB disease. In all analysis strategies, UV lights tended to be more protective than ionizers.

**Conclusions:**

Upper-room UV lights and negative air ionization each prevented most airborne TB transmission detectable by guinea pig air sampling. Provided there is adequate mixing of room air, upper-room UV light is an effective, low-cost intervention for use in TB infection control in high-risk clinical settings.

## Abstract 2

Using a guinea-pig detection model, Rod Escombe and colleagues find that upper-room UV lamps in hospital rooms can substantially reduce airborne transmission of*Mycobacterium tuberculosis*.

## Editors' Summary

**Background.:**

Tuberculosis—a contagious infection, usually of the lungs—kills nearly 2 million people annually. It is caused by Mycobacterium tuberculosis, bacteria that are spread in airborne droplets when people with tuberculosis cough or sneeze. Most people infected with M. tuberculosis do not become ill—their immune system contains the infection. However, the bacteria remain dormant within the body and can cause disease years later if immunity declines because of, for example, infection with human immunodeficiency virus (HIV), the cause of acquired immunodeficiency syndrome (AIDS). The symptoms of tuberculosis include a persistent cough, weight loss, and night sweats. Infection with M. tuberculosis is diagnosed using the tuberculin skin test. Tests for tuberculosis itself include chest X-rays and sputum cultures (in which bacteriologists try to grow M. tuberculosis from mucus brought up from the lungs by coughing). Tuberculosis can usually be cured by taking several powerful antibiotics daily for several months. Drug-resistant tuberculosis is much harder to cure, requiring multiple second-line antibiotics for up to two years or more. Tuberculosis transmission can be reduced by, for example, hospitalizing people with tuberculosis in isolation wards in which negative-pressure mechanical ventilation is used to reduce the concentration of infectious airborne droplets.

**Why Was This Study Done?:**

After the development of antibiotics capable of killing M. tuberculosis in the mid 20th century, it seemed that tuberculosis would become a disease of the past. But in the mid 1980s, drug-resistant M. tuberculosis strains began to emerge, the HIV/AIDS epidemic took hold, and tuberculosis resurged to today's worrying levels. New ways of reducing tuberculosis transmission, particularly in health care settings and in resource-limited settings, are now urgently needed. The need for effective infection control measures is especially urgent in HIV care programs where highly susceptible individuals frequently mix with people with tuberculosis. In this study, the researchers use a guinea pig air-sampling model (which was first used in the 1950s to show that tuberculosis is an airborne infection) to investigate whether upper-room ultraviolet (UV) lights in patient rooms and negative air ionization can prevent airborne tuberculosis transmission. UV light kills M. tuberculosis; negative ionization gives airborne particles a charge that makes them stick to surfaces.

**What Did the Researchers Do and Find?:**

The researchers exposed a group of control guinea pigs kept in a special air-sampling enclosure to untreated air from an HIV–TB ward in Lima (Perú). Another group of animals (the UV group) breathed air from the same ward, but only on the days that UV lights suspended near the ward's ceiling were turned on, together with mixing fans to mix up the room air. The “ionizer group” had a negative ionizer switched on in their enclosure when they were exposed to ward air (each group of animals was exposed to ward air every other day). The animals were tested monthly with the tuberculin skin test and all were examined for tuberculosis disease when they became infected with tuberculosis or at the end of the 535-day experiment. 35% of the control animals, 14% of the ionizer group animals, and 9.5% of the UV group animals developed M. tuberculosis infections. Tuberculosis disease was found in 8.6% of the control animals but in only 4.3% and 3.6% of the animals in the ionizer and UV groups, respectively. A “time-to-event analysis” also showed that UV lights and ionizers reduced tuberculosis infection and disease. Finally, an analysis of the data using an airborne infection model indicated that ionizers and UV lights prevented 60% and 70% of tuberculosis infections, respectively.

**What Do These Findings Mean?:**

These findings indicate that upper-room UV lights, combined with adequate air mixing, or negative air ionization with special large-scale ionizers can prevent most airborne tuberculosis transmission to guinea pigs exposed to hospital room air. The effectiveness of these approaches in reducing tuberculosis transmission between people is likely to be similar, although remains to be tested. Nevertheless, this first study of the effect of upper-air UV light and of negative air ionization on airborne transmission in a clinical setting suggests that both approaches could be potentially important tuberculosis infection control measures. Furthermore, the UV light approach might provide a relatively low-cost intervention for possible use in waiting rooms and other overcrowded settings where patients with undiagnosed, untreated tuberculosis—individuals who tend to be highly infectious—are likely to come into contact with other susceptible patients, health care workers, and visitors.

**Additional Information.:**

Please access these Web sites via the online version of this summary at http://dx.doi.org/10.1371/journal.pmed.1000043.

- The US National Institute of Allergy and Infectious Diseases provides information on all aspects of tuberculosis, including multidrug-resistance tuberculosis, and on tuberculosis and HIV
- The US Centers for Disease Control and Prevention provide several fact sheets and other information resources about all aspects of tuberculosis, including Guidelines for preventing the Transmission of *Mycobacterium tuberculosis* in Health-Care Settings, 2005 (some information in Spanish is also available)
- The World Health Organization's 2008 report “Global Tuberculosis Control—Surveillance, Planning, Financing” provides a snapshot of the current state of the global tuberculosis epidemic and links to information about all aspects of tuberculosis and its control (in several languages)
- Tuberculosis Infection-Control in the Era of Expanding HIV Care and Treatment is another report from the World Health Organization
- HIVInsite provides detailed information about the combination of HIV infection and tuberculosis
- Avert, an international AIDS charity, also provides information about the interaction between HIV and tuberculosis
- GHD (Global Health Delivery) Online is an online resource dedicated to TB infection control, and is moderated by world experts

## Introduction

Tuberculosis (TB) infection control remains a public health priority, especially with the emergence of extensively drug-resistant strains [1]. TB outbreaks have long been reported in congregate settings [2] including hospitals [3,4], homeless shelters [5], and correctional facilities [6]. Nosocomial transmission and occupational TB are common in resource-limited settings, especially where TB and HIV are prevalent [7–9]. The expansion of HIV care programmes may inadvertently increase TB transmission by congregating highly susceptible individuals with those likely to have TB disease [10].

Guidelines for preventing TB transmission in health care settings advocate administrative control measures to ensure prompt diagnosis, isolation, and treatment of TB patients; environmental control measures to reduce the concentration of airborne infectious droplet nuclei; and personal respiratory protection [11,12]. Environmental control measures such as negative-pressure mechanical ventilation are expensive to install and maintain, and offer limited protection [13]. Natural ventilation may provide greater protection for little cost [14], but is climate dependent. Additional environmental control measures are urgently needed.

Upper-room ultraviolet (UV) light is recommended for reducing TB transmission in health care facilities [12,15]. However, despite renewed research interest [16,17], its use is not widespread due mainly to the absence of efficacy studies in clinical settings. Negative air ionizers charge airborne particles resulting in attraction to grounded surfaces and clearance from the air. They also have bactericidal effects [18–20]. Large-scale negative ionizers successfully reduced airborne *Salmonella* transmission by up to 99% in poultry hatching [21–24]. We hypothesized a novel effect of ionizers in preventing airborne TB transmission.

An accepted strategy for detecting viable airborne M. tuberculosis in clinical areas is air sampling using guinea pigs [25], which are extremely susceptible to TB. Classic 1950s studies utilizing a guinea pig air-sampling facility on the roof of a TB ward defined the airborne nature of TB transmission, and demonstrated the germicidal efficacy of unshielded UV lights inside air ducts [26,27]. We extended this experimental model in order to evaluate upper-room UV light and negative air ionization for preventing TB transmission.

## Methods

### Setting

**Figure 1:**
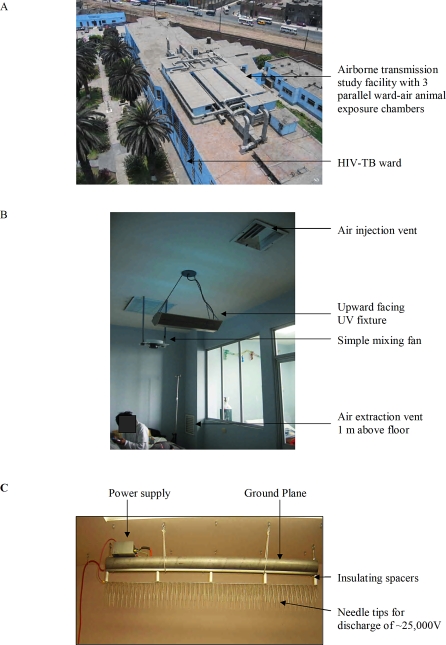
Bird's Eye View of Guinea Pig Air Sampling Facility on Roof of HIV-TB Ward; Photograph of HIV-TB Isolation Room Showing UV Light Fixture, Mixing Fan, and Ventilation System; and Photograph of Large-Scale ESCS Ionizer (A) Airborne transmission study facility on the roof of HIV-TB ward. Negative-pressure respiratory isolation rooms for TB-HIV co-infected patients were located on the ground floor, of which four were used in the study. All air from these rooms was ducted to the roof, where it was either passed over different groups of guinea pigs in one of three parallel enclosures (exposure chambers), or was sterilised and exhausted to the environment through the chimneys. (B) Patient isolation room with upper-room UV light fixture. The design of the UV fixture with baffling of the UV bulbs restricts the high intensity UV light to the upper part of the room. Each fixture had two 9-W UV bulbs. Adjacent to the fixture is a simple 382-m^3^/h fan used to assist mixing of lower- and upper-room air. The mechanical ventilation system's air injection vent in the ceiling and the air extraction vent at bed height (1 m) are also shown. (C) ESCS. An ESCS ionizer suspended from the ceiling is shown. Approximately −25,000 V were delivered to ∼200 needle tips, where negative ions are generated in adjacent air due to corona discharge. The current was limited to <1 mA to ensure personal safety. Three ESCS ionizers were sited in the Ionizer animal enclosure.

The HIV-TB ward at Hospital Nacional Dos de Mayo, Lima, Perú was converted into eight negative-pressure isolation rooms. Four were used in the study. Air injection vents in the ceiling and air extraction vents close to the head of a patient lying in bed were positioned based on computational fluid dynamics (CFD) modelling of the ventilation in patient rooms [28]. The exhaust vents were positioned in the lower part of the room to ensure that the guinea pigs sampled the air breathed by room occupants without it being deliberately drawn through the UV field. A new airborne TB-transmission study facility was constructed on the roof (Figure 1A). Three parallel negatively pressurised 70-m^3^ exposure chambers each housed up to 150 guinea pigs and were ventilated on alternate days with ward air, or fresh outside air. Animal cages had wire mesh floors to minimize the risk of faecal-oral TB transmission. Cages were isolated by partitions to limit any horizontal disease outbreak to the six animals per cage.

### Patient Recruitment

HIV-infected patients with pulmonary TB were admitted to the negative-pressure rooms and invited to join the study with written informed consent. Patient admission, management, or duration of hospital stay was uninfluenced by the study. Sputum smear-positive patients were preferentially located in the study rooms, from which air passed to the air-sampling facility on the roof. Sputum samples were collected on admission and weekly thereafter for auramine staining and TB culture using MODS [29]. Patients occupied their rooms 24 h a day, generally only leaving for activities such as X-rays.

### UV Lights

An upward-facing UV light fixture (Lumalier) (Figure 1B) was suspended from the ceiling in each room with a small fan adjacent to facilitate room air mixing. UV fixtures were baffled to deliver UV intensity ≤0.4 μW/cm^2^ at a height of 1.8 m. Bulbs were cleaned with alcohol every 2 mo, and UV fields in the room were remeasured after cleaning. Health care staff and patients sequentially wore a data-logging personal UV-exposure meter (Gigahertz-Optik) and completed questionnaires about adverse UV effects.

### Negative Air Ionization

Electrostatic space charge systems (ESCSs; prototype large-scale negative ionizers [Figure 1C] [22,23]) were selected due to their high negative ion generation rate following testing and comparison with 24 commercially available negative ionizers, all of which performed poorly (unpublished data). Three ESCS ionizer units were ceiling-mounted inside the third animal enclosure and functioned continuously. Negative ion concentration was monitored indirectly using a charge-decay meter (IPA-287; Monroe Electronics). Ozone levels were monitored (Aeroqual500; Ozone Solutions).

### Experimental Protocol

Out-bred Peruvian guinea pigs maintained in quarantine were skin tested monthly with 100 units purified protein derivative (PPD; Evans-Vaccines) [30] and induration diameter was measured 24 h later. Animals were transferred to the study facility following ≥2 negative skin tests to ensure freedom from TB infection. Up to 150 animals were maintained in each ward-air exposure chamber, designated “Control,” “UV,” and “Ionizers.” Monthly skin tests were continued with reading performed by the same operator, blinded to animal group. A ≥7.5-mm cut-off was used for a positive PPD test to indicate TB infection as described [30]. PPD-positive animals and those losing >200 g weight were removed monthly for sacrifice, autopsy, and organ culture for M. tuberculosis as described to detect TB disease [30]. Additional animals were sacrificed monthly, such that equal numbers were removed from each chamber. Sacrificed animals were replaced with new quarantined animals. A cohort of negative control animals exposed to fresh air alone was maintained separately.

### Rotation of Air

**Figure 2:**
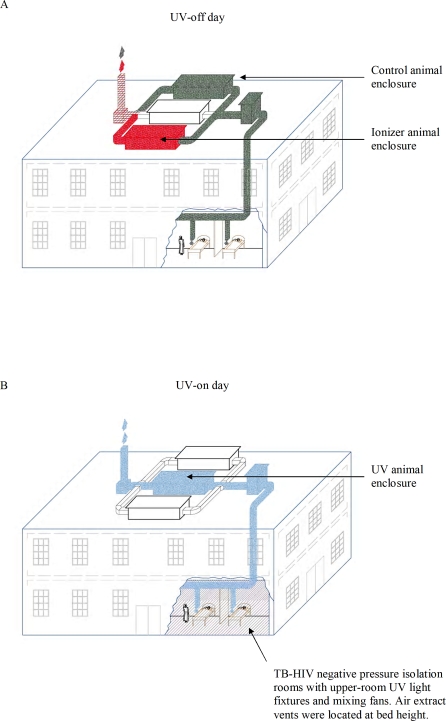
Schematic of the Airborne TB Transmission Study Facility on the Roof of an HIV-TB Ward in Lima, Perú All exhaust air from the four patient isolation rooms passed over the guinea pigs, housed in groups of up to 150 animals in three exposure chambers: Control group (green); UV lights group (blue); and Ionizer group (red). Guinea pigs were exposed on alternate days to ward air or fresh outside air, according to the rotation below, with a 45-min wash-out period between study days. (A) UV-off day (Control day): No air cleaning intervention on the ward. Exhaust air from the ward was divided on the roof, half passing through the Control enclosure of guinea pigs, and half passing through the Ionizer enclosure, inside which were located three large space negative air ionizers (ESCSs). On these days, fresh outside air was passed through the centrally positioned UV lights group exposure chamber. (B) UV-on day: UV lights and mixing fans were turned on in the ward. Half of the ward air passed through the UV lights group animal enclosure, and half was sterilised and exhausted into the atmosphere. The other two exposure chambers were ventilated with fresh outside air.

Guinea pig enclosures were ventilated on alternate days with ward air or fresh air. When ventilated with ward air, this air was untreated for the Control group, UV-treated for the UV group, and ionized for the Ionizer group. To achieve this, a 2-day rotation was used: UV-on days and UV-off days (Figure 2). On UV-on days, UV lights on the ward were turned on continuously (together with mixing fans during daylight hours). Correct functioning of lights and fans was confirmed daily by visual inspection. On these UV-on days, half the exhaust air from the ward was passed through the UV animal enclosure and the other half was sterilised and exhausted outside, and the other two animal enclosures were ventilated with fresh outside air. On UV-off days, UV lights were switched off and the ward air was divided so that half passed through the Control enclosure and half through the Ionizer enclosure. Thus, the Control group breathed ward air without any infection control intervention; the UV group breathed ward air only when the ward UV lights were on; and the Ionizer group breathed ward air treated with ionizers (but not UV lights). A 45-min purge period separated study days allowing replacement of partially UV-treated ward air with fresh air after UV-on days. Study “days” were therefore 23 h and 15 min long. Airflow leaving the ward, and into and out of each animal enclosure was balanced, and measured daily using in-duct iris dampers (Continental Fan Inc.).

### Statistics

Statistical analysis was performed using SPSS v10.0. Both TB infections (measured as numbers of PPD positive conversions), and TB disease (defined as autopsy or culture evidence of TB disease) were compared between groups using two samples proportions tests for numbers of animals in each group. To allow for minor differences between groups in numbers of animals and total exposure time, time-to-event was also analyzed for TB infection or disease using the log-rank test and are shown as Kaplan-Meier plots, censored when group sizes fell to ten animals [31]. Mean airflow data between animal enclosures were compared using analysis of variance. An alternative analysis was performed using the Wells-Riley airborne infection model [15] to determine the overall effect of the two interventions on TB transmission measured as both TB infection and TB disease in guinea pigs, accounting for minor airflow differences between the animal enclosures as well as seasonal airflow variation (see Text S1). Room air mixing was estimated using computational fluid dynamics modelling [28], and a separate zonal mixing model was used to predict upper-room UV effectiveness (see Text S1). All *p*-values are two-sided.

### Ethical Approval

The study was approved by the Institutional Review Boards at Hospital Dos de Mayo, Asociación Benéfica PRISMA, and Imperial College London Hammersmith Hospital Campus. The Veterinary Faculty, Universidad Nacional Mayor San Marcos, granted animal ethical approval and supervised animal work.

## Results

### Patients

Over 535 study days, 69 HIV-positive patients with pulmonary TB made a total of 80 ward admissions. 59 (74%) admissions were by smear-positive patients. Median hospital stay was 9 d (interquartile range [IQR] 5–17 d). There were 612 (50.3%) and 606 (49.7%) patient bed days on UV-off and UV-on study days, respectively.

### Airflow, UV-Light, and Ionizer Monitoring

Ward ventilation was maintained at 5.9 (standard deviation, SD 0.4) air-changes/hour. Room air was well mixed (see Text S1). There was no significant difference between the volume of ward air received by each animal group (ANOVA; *p =* 0.1). Although the difference in mean extracted air was significant between groups (ANOVA; *p =* 0.001), this difference was less than 4%. Mean fresh air infiltration into Control, UV, and Ionizer enclosures caused by leakage into the negative-pressure system was 9.2, 14, and 12 cfm, respectively, representing 4.1%–6.3% of the air received by the animals. There were no patient or staff UV-related complaints, and UV exposure was within recommended limits [12]. Mean ward UV intensity at height 1.8 m diminished by 6.5% during the study. In the Ionizer enclosure, high negative ion concentration was demonstrated using a +1,050-V charged plate located 1 m from each ESCS ionizer. This plate consistently discharged to zero within 5 s. The maximum ozone level detected was 0.06 ppm. Median ward temperature was 19.2 °C (IQR 17.8–22.5 °C) and median relative humidity 77% (IQR 73%–80%). Differences in mean temperature and mean relative humidity between animal enclosures were small (<0.2 °C and <2%, respectively), and there were no significant differences in these parameters between UV-on and UV-off days (ANOVA; *p >* 0.3 in all cases).

### Detection of TB in Guinea Pigs

**Figure 3:**
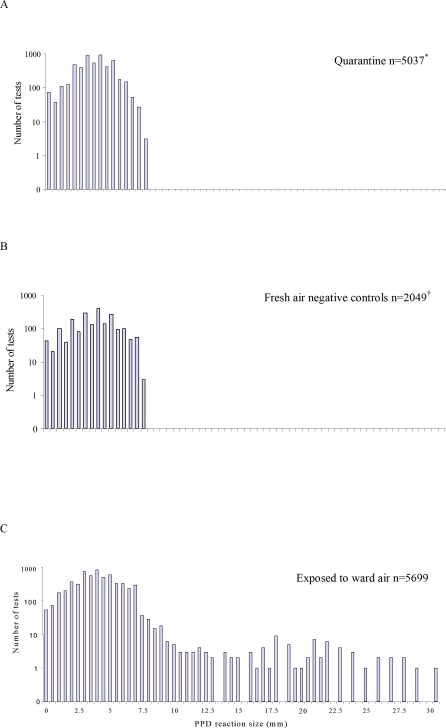
Guinea Pig PPD Skin Test Responses The frequency distributions of guinea pig PPD skin test responses are shown for (A) quarantined animals, (B) roof air negative controls, and (C) ward-air exposed guinea pigs. The frequencies of the PPD responses have been plotted using a logarithmic scale on the *y*-axis to demonstrate the distribution of positive (≥7.5 mm) responses seen in the exposed group of animals. Zero frequencies are plotted on the logarithmic scale as zero. PPD responses were measured to the nearest 0.5 mm; therefore, each bar represents the frequency of a single measurement value. *In 5,037 tests in 624 animals over 11 mo, there were three false positive PPD skin test results in quarantine animals. All were 7.5 mm and reverted to <7.5 mm in subsequent months. †In 2,049 tests in fresh-air negative controls, there was one false positive result (7.5 mm).

PPD skin test responses for quarantine, negative control, and ward-air exposed guinea pigs are shown (Figure 3). There was no evidence of boosting with serial testing. In 5,699 skin tests in ward-air exposed animals, there were 109 animals with TB infection in the Control group, 53 in the Ionizer group, and 30 in the UV group resulting from 62,844, 62,921, and 63,170 animal exposure days, respectively.

### Dust-Related TB Outbreak

**Figure 4:**
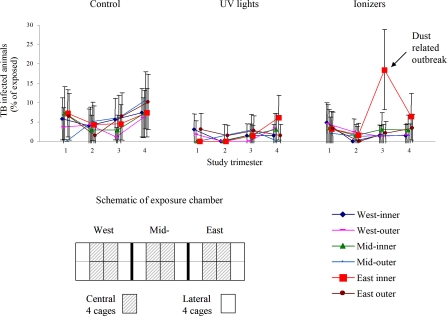
Distribution Over Time of TB-Infected Animals According to Cage Location Demonstrating Dust-Related TB Outbreak A schematic is shown of one of the three identical guinea pig exposure chambers, each with 24 cages arranged in two rows. Each chamber is divided into three sections (east, mid, and west). Thus each section has eight cages, four located centrally and four located laterally within each section. TB-infected animals have been grouped in 3-mo study periods (trimesters), excluding the first and last 2 mo of the study owing to small numbers as the study started and finished. The third trimester immediately followed the cleaning of an ionizer creating a dust cloud directly above the four centrally located cages in the east section of the ioniser animal enclosure. Numbers of TB-infected animals (expressed as a percentage of total numbers of animals exposed) are shown for each of the six parts of the Control, UV, and Ionizer guinea pig exposure chambers, for three-monthly study periods.

In month 7 during ionizer cleaning a cloud of thick soot-like dust that had collected on the ionizer scattered over adjacent animal cages in the east-central part of the animal enclosure (Figure 4). In subsequent skin tests (months 8–10), there was a significant increase in the proportion of TB-infected animals in these cages compared with surrounding cages and with previous months (two samples proportions test; *p <* 0.0001; Figure 4). Subsequent cleanings were undertaken more carefully, spraying first with bleach. To avoid confounding by this artefactual TB transmission from ionizer cleaning, all animals in the east-central cages of all exposure chambers for tests 8–10 were excluded from analyses (constituting three, one, and ten TB-infected animals for Control, UV, and Ionizer groups, respectively, which for time-to-event analyses were censored using the seventh skin test date; one of these animals, in the Ionizer group, had evidence of TB disease). Spatial distribution of TB infections in the exposure chambers was otherwise random, implying airborne transmission from the ward and not horizontal transmission between animals. Culture for TB of dust-like particulates from the ceiling and the ESCS ionizer was negative.

### Reduction of Airborne TB Transmission—Proportions Analysis

**Figure 5:**
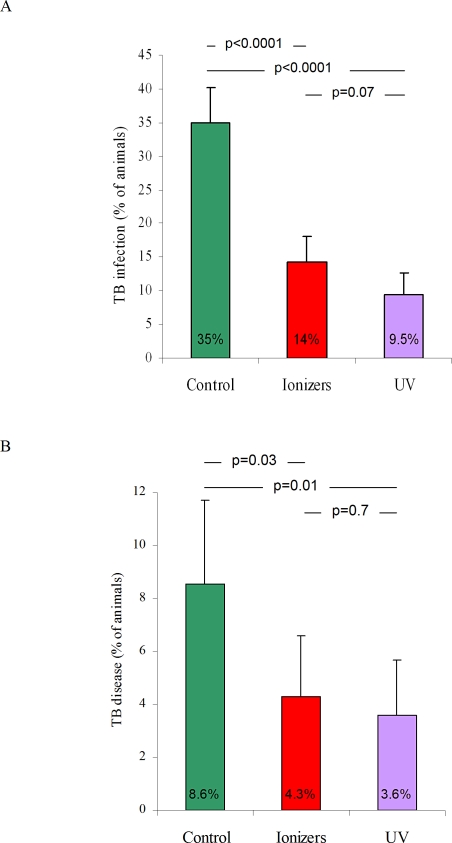
Proportion of Ward-Air Exposed Animals in Each Experimental Group with Evidence of TB Infection or TB Disease (A) TB infection (PPD skin test conversions). (B) TB disease (autopsy or culture evidence of TB).

Having excluded the dust outbreak animals as above, after 535 study days there were 106/304 (35%) animals with TB infection in the Control group, 43/303 (14%) in the Ionizer group, and 29/307 (9.5%) in the UV group (two samples proportions test: Control versus Ionizers and Control versus UV both *p <* 0.0001; Ionizers versus UV *p =* 0.07; Figure 5A). Autopsy and TB culture results for PPD-positive animals, PPD-negative animals, and intercurrent deaths are shown (Table 1). TB disease was confirmed in 26/304 (8.6%) animals in the Control group, 13/303 (4.3%) animals in the Ionizer group, and 11/307 (3.6%) animals in the UV group (two samples proportions test: Control versus Ionizers *p =* 0.03; Control versus UV *p =* 0.01; Ionizers versus UV *p =* 0.65; Figure 5B). Confirmatory analysis of these TB infection and TB disease proportions with the Chi-squared test to reject the null-hypothesis with post hoc Marascuillo procedure to allow for multiple comparisons confirmed this pattern of significance.

**Table 1:**
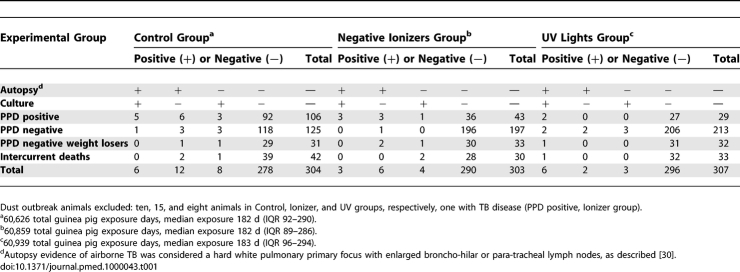
PPD Skin Test, Autopsy, and Culture Results for Ward-Air Exposed Guinea Pigs

### Reduction of Airborne TB Transmission—Time-to-Event Analysis

**Figure 6:**
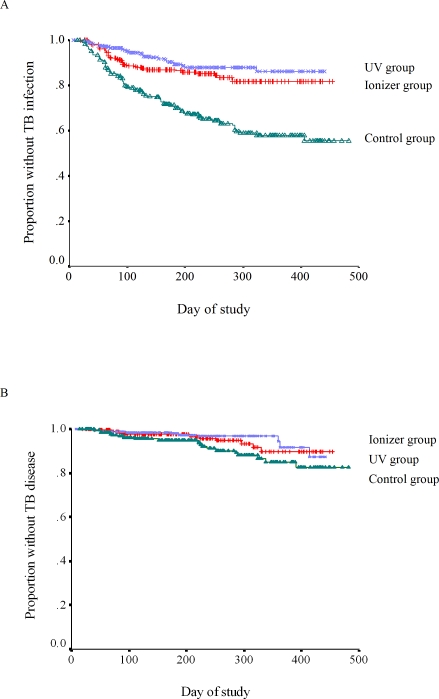
Kaplan-Meier Time-to-Event Curves (A) Using TB infection (PPD skin test conversion) as the end-point. (B) Using TB disease (autopsy or culture evidence of TB) as the end-point.

Time-to-event analysis demonstrated that Ionizers and UV lights were both significantly protective against TB infection (log-rank 27; *p <* 0.0001 and log-rank 46; *p <* 0.0001, respectively; Figure 6A). Similarly, time-to-event analysis demonstrated that Ionizers and UV lights were both protective against TB disease, (log-rank 3.7; *p =* 0.055 and log-rank 5.4; *p =* 0.02, respectively; Figure 6B). Time-to-event analysis demonstrated that there was a nonsignificant trend for UV lights to be more protective than ionizers against TB infection and disease (log rank 2.9; *p =* 0.09 and log rank 0.2; *p =* 0.6, respectively).

### Reduction of Airborne TB Transmission—Airborne Infection Model Analysis

The alternative Wells-Riley analysis demonstrated 60% and 70% reductions in TB infections because of ionizers and UV lights, respectively, after correcting for minor airflow differences between groups. Comparing this with the differences across the three groups prior to correcting for minor airflow variation, suggests that in both cases a further ∼1% reduction in TB infections was due to the small differences in airflow between the groups. The zonal mixing model predicted upper-room UV to cause a reduction of approximately 79% in the concentration of airborne infectious particles in the lower part of the room (see Text S1). The Wells-Riley analysis demonstrated 51% and 54% reductions in TB disease due to ionizers and UV lights, respectively.

Further analyses using the three methodologies above were performed with the “dust outbreak” animals included. All results were similar, except that the protective effect of Ionizers against TB disease compared with the Control group no longer reached the traditional threshold for statistical significance in time-to-event analysis (log-rank 3.1; *p =* 0.08).

## Discussion

This is, to our knowledge, the first controlled evaluation of the effect of upper-room UV light or negative air ionization on airborne TB transmission in a clinical setting. By using a guinea pig air-sampling model to measure the TB infectiousness of air, we have demonstrated that both interventions prevented most TB transmission and are therefore potentially important TB infection control measures.

On the basis of laboratory evidence of mycobacterial susceptibility to UV light [32], the effects of UV lamps on mycobacterial aerosols [16,17,33], modelling studies [34–36], and anecdotal evidence from hospitals with UV lights [37,38], the U.S. Centers for Disease Control and Prevention recommends upper-room UV light as an environmental control measure for TB in health care settings [12]. However, use is not widespread, owing primarily to the lack of studies in a clinical setting. Upper-room UV in a hospital waiting room reduced airborne bacteria, but TB transmission was not investigated due to the logistical difficulties such studies entail [39]. The efficacy of UV light demonstrated in the current study has important implications for TB infection control, especially in low-resource settings, where the burden of TB is highest. Upper-room UV light is a relatively low-cost intervention compared with mechanical ventilation. Furthermore, it is ideally suited to overcrowded congregate settings such as waiting rooms, out-patient and emergency departments, or anti-retroviral treatment facilities. Such areas rarely have high air-exchange mechanical ventilation, but represent a TB infection control priority because undiagnosed and untreated TB patients are likely to be found there, and these are generally the most infectious TB patients.

In addition to the absence of efficacy studies in a clinical setting, safety concerns have discouraged use of upper-room UV light. However, appropriate positioning of modern, shielded UV fixtures allows the upper room to be flooded with high-intensity UV light whilst minimizing occupant exposure in the lower part of the room. Previously reported cases of over-exposure resulted from incorrect lamp installation or inadequate action if a UV bulb became unshielded [40,41]. Expert installation of upper-room UV systems is therefore imperative, with postinstallation checking of UV fields to ensure there is no excess UV light reflected downwards. Indeed following lamp installation in this study, bulbs required additional baffles and the ceiling was painted matt to reduce reflections. No adverse UV effects were reported by patients or staff, and staff and patient exposures measured with the personal UV meter were within permitted levels [12]. This concurs with other recent data concerning upper-room UV safety [42,43]. Manufacturers may recommend replacing bulbs annually, but during this study UV intensity decreased minimally following >6,000 h use, despite daily on/off switching, which degrades UV bulbs quicker. An irradiance decline limit of 30% has been suggested whilst upper-room UV installation and maintenance guidelines remain under development [44]. Proactive maintenance, including a planned programme for bulb replacement, allows adequate UV fields to be maintained before bulb failure occurs. Despite experience gained using upper-room UV in 14 homeless shelters [42], further operational research is needed to assess limitations, such as the effect of periodic power outages, and the creation of a false sense of security. The current cost of the fixtures in this study is US$355–709 including bulbs, the lower range for export to low-resource settings. Replacement bulbs cost US$25–50. Considerable potential exists for developing lower cost UV fixtures for TB control in resource-limited settings.

Upper-room UV light efficacy depends on adequate mixing of lower- and upper-room air through simple convection currents [45,46] that may be augmented by mechanical ventilation systems, or inexpensive mixing fans [17,47], as in the current study. Air was well mixed in the patient rooms in this study as a result of the mechanical ventilation system delivering 236 m^3^/h of fresh air through a ceiling vent, the mixing fan adjacent to the UV fixture, and other air currents generated by convection, movement of room occupants, and door opening. Computational fluid dynamics modelling together with mixing fan specifications allowed room air to be estimated as 83% mixed, and the zonal mixing model for estimating upper-room UV efficacy predicted a 79% reduction in the concentration of infectious particles in the lower room. This figure correlates well with the 70% reduction in TB infections actually observed in the guinea pigs. The presence of good air mixing in the patient rooms therefore allows extrapolation of the results for the protection of guinea pigs from airborne TB infection to the protection of room occupants. However, upper-room UV will never be able to protect a health care worker from airborne infection occurring at very close proximity. Controlled studies of the effect of upper-room UV on human-to-human TB transmission are warranted. However, studies of TB infection in health care workers are logistically challenging due to staff turnover, and confounded by TB exposures in other parts of a health care facility, or exposures outside the workplace.

UV germicidal efficacy is adversely affected by increasing relative humidity, particularly above 50%–60% [17]. Relative humidity in Lima is generally 70%–90%, and the hospital rooms studied were not air conditioned. It was therefore unexpected to see such a marked effect of upper-room UV light on TB transmission. This protective effect of UV lights against TB transmission may be greater in less humid environments, including the air-conditioned environments of hospitals in resource-rich settings.

This is the first published evaluation of negative air ionization for preventing TB transmission, to our knowledge. The mechanism is most likely a physical effect of charged airborne particulates being strongly attracted to grounded surfaces such as walls and floors. A bactericidal effect is also possible [18,20]. Reductions in airborne transmission in this study were less than those observed in chick hatcheries and production areas installed with ESCS ionizers where transmission of *Salmonella* was reduced by up to 99.8% [21–24]. It is possible higher ion densities were achieved in the poultry studies or that slower air movement allowed greater airborne particle removal, and ionizer effectiveness decreases as relative humidity increases.

A disadvantage to negative air ionization is the accumulation of potentially infectious particles onto adjacent surfaces or grounded parts of the ionizer itself, as suggested by the localized outbreak of TB-infected animals following ionizer cleaning. The walls of the ionizer exposure chamber quickly became discoloured, the “black-wall effect” seen with some commercially available ionizers. These problems may be mitigated using localized grounded collecting plates. Another potential disadvantage of ionizers is static charge interference with medical equipment, although they have been used for several years in an intensive care unit without reported problems [48]. The static charge effect on instrumentation is minimal beyond 1 m from the ionizer. The prototype ESCS ionizer used in this study has been shown to require careful handling and cleaning, and is therefore not yet appropriate for clinical use, particularly in low-resource settings. However, improved ESCS ionizers with increased range and effectiveness, and fewer needles to facilitate easier cleaning have already been developed and commercialized for agricultural applications, and these devices warrant further study. Approximate ESCS ionizer costs are US$600 for an isolation room, US$300 for subsequent rooms, and the intrinsically simple components facilitate the potential for inexpensive manufacture.

One limitation of this study is that the animal facility was not tested with artificial aerosols to evaluate airflow patterns and particle loss between ward and roof. However, careful ventilation system balancing during commissioning and daily airflow measurement at multiple locations demonstrated only small differences in ward airflow or outside air infiltration into the three exposure chambers. Any particle loss between ward and animal chambers would be expected to be similar on UV-on or UV-off days. Another limitation is that ionizers were located on the roof to avoid the potential artefact of negatively charged droplet nuclei attaching to lengthy metal ductwork between ward and animal facility. The ionizer effect on TB transmission in the ward itself may have differed to that observed in the animal facility.

The finding of large numbers of PPD-positive animals without autopsy or culture evidence of TB was initially surprising, but is consistent with previous findings if the bias of two outbreaks of a highly infectious, highly virulent strain are accounted for [30,49]. Experimental airborne infection of guinea pigs with clinical isolates of M. tuberculosis has demonstrated that up to four colony forming units are required to establish disease [50]. It is likely that PPD-positive but autopsy-negative animals were infected with doses insufficient to establish disease, or with strains poorly virulent for guinea pigs. No PPD responses >7.5 mm were observed in over 7,000 tests in quarantine and negative control animals, making PPD conversion almost certainly due to TB infection. There was no increased proportion of PPD-positive, autopsy-, and culture-negative animals seen in the UV group, which might have been expected, were PPD conversion to occur from exposure to dead or UV-damaged mycobacteria. These interesting data suggest the need for additional studies that may characterize true latent TB infection versus delayed hypersensitivity alone, or the possibility of transient TB infection [51].

TB infection control is a global public health priority owing to the HIV epidemic, and to the importance of nosocomial transmission in the propagation of multi- and extensively drug-resistant strains. The expansion of HIV care, entailing congregation in overcrowded settings of highly susceptible persons with those most likely to have TB, accentuates the need for effective prevention of TB transmission. Modelling studies of the XDR-TB outbreak in South Africa have suggested that administrative control measures may be inadequate [52], and therefore environmental control measures assume increasing importance. Upper-room UV light is an effective and relatively low-cost environmental control measure already recommended as an adjunctive measure by guidelines, but little used owing to the lack of studies in clinical settings until now [12]. Upper-room UV has great potential for TB infection control in settings where climate does not permit the use of natural ventilation, or for use in cooler winter months, or at night when windows are likely to be closed. Efforts should be made to design simple UV fixtures for low-resource settings. Negative air ionization is a novel infection control strategy warranting further investigation. These environmental control measures are well suited to large congregate settings such as waiting rooms or emergency departments, where TB is often undiagnosed, overcrowding is common, and respiratory isolation facilities are frequently unavailable.

## Supporting Information

Text S1 Wells-Riley Calculation Methodology and Room Air Mixing and UV Lamp Effectiveness Schematic (74 KB DOC)

## Abbreviations

ESCS: electrostatic space charge system
IQR: interquartile range
PPD: purified protein derivative
TB: tuberculosis
UV: ultraviolet

## References

[pmed-1000043-b001] Gandhi NR, Moll A, Sturm AW, Pawinski R, Govender T (2006). Extensively drug-resistant tuberculosis as a cause of death in patients co-infected with tuberculosis and HIV in a rural area of South Africa. Lancet.

[pmed-1000043-b002] Mohle-Boetani JC, Miguelino V, Dewsnup DH, Desmond E, Horowitz E (2002). Tuberculosis outbreak in a housing unit for human immunodeficiency virus-infected patients in a correctional facility: transmission risk factors and effective outbreak control. Clin Infect Dis.

[pmed-1000043-b003] Catanzaro A (1982). Nosocomial tuberculosis. Am Rev Respir Dis.

[pmed-1000043-b004] Alland D, Kalkut GE, Moss AR, McAdam RA, Hahn JA (1994). Transmission of tuberculosis in New York City. An analysis by DNA fingerprinting and conventional epidemiologic methods. N Engl J Med.

[pmed-1000043-b005] Dwyer B, Jackson K, Raios K, Sievers A, Wilshire E (1993). DNA restriction fragment analysis to define an extended cluster of tuberculosis in homeless men and their associates. J Infect Dis.

[pmed-1000043-b006] Valway SE, Greifinger RB, Papania M, Kilburn JO, Woodley C (1994). Multidrug-resistant tuberculosis in the New York State prison system, 1990–1991. J Infect Dis.

[pmed-1000043-b007] Pai M, Kalantri S, Aggarwal AN, Menzies D, Blumberg HM (2006). Nosocomial tuberculosis in India. Emerg Infect Dis.

[pmed-1000043-b008] Joshi R, Reingold AL, Menzies D, Pai M (2006). Tuberculosis among health-care workers in low- and middle-income countries: a systematic review. PLoS Med.

[pmed-1000043-b009] Menzies D, Joshi R, Pai M (2007). Risk of tuberculosis infection and disease associated with work in health care settings. Int J Tuberc Lung Dis.

[pmed-1000043-b010] Bock N, Jensen P, Miller B, Nardell E (2007). Tuberculosis infection control in resource limitied settings in the era of expanding HIV care and treatment. J Infect Dis 196 Suppl.

[pmed-1000043-b011] World Health Organisation (1999). Guidelines for the prevention of tuberculosis in healthcare facilities in resource-limited settings.

[pmed-1000043-b012] Jensen PA, Lambert LA, Iademarco MF, Ridzon R (2005). Guidelines for preventing the transmission of Mycobacterium tuberculosis in health-care settings, 2005. MMWR Recomm Rep.

[pmed-1000043-b013] Nardell EA, Keegan J, Cheney SA, Etkind SC (1991). Airborne infection. Theoretical limits of protection achievable by building ventilation. Am Rev Respir Dis.

[pmed-1000043-b014] Escombe AR, Oeser CC, Gilman RH, Navincopa M, Ticona E (2007). Natural ventilation for the prevention of airborne contagion. PLoS Med.

[pmed-1000043-b015] Riley RL, Nardell EA (1989). Clearing the air. The theory and application of ultraviolet air disinfection. Am Rev Respir Dis.

[pmed-1000043-b016] Xu P, Peccia J, Fabian P, Martyny JW, Fennelly KP (2003). Efficacy of ultraviolet germicidal irradiation of upper-room air in inactivating bacterial spores and Mycobacteria in full-scale studies. Atmos Environ.

[pmed-1000043-b017] Xu P, Kujundzic E, Peccia J, Schafer MP, Moss G (2005). Impact of environmental factors on efficacy of upper-room air ultraviolet germicidal irradiation for inactivating airborne mycobacteria. Environ Sci Technol.

[pmed-1000043-b018] Seo KH, Mitchell BW, Holt PS, Gast RK (2001). Bactericidal effects of negative air ions on airborne and surface Salmonella enteritidis from an artificially generated aerosol. J Food Prot.

[pmed-1000043-b019] Arnold JW, Mitchell BW (2002). Use of negative air ionization for reducing microbial contamination on stainless steel surfaces. J Appl Poult Res.

[pmed-1000043-b020] Fletcher LA, Gaunt LF, Beggs CB, Shepherd SJ, Sleigh P (2007). Bactericidal action of positive and negative ions in air. BMC Microbiol.

[pmed-1000043-b021] Richardson LJ, Hofacre CL, Mitchell BW, Wilson JL (2003). Effect of electrostatic space charge on reduction of airborne transmission of Salmonella and other bacteria in broiler breeders in production and their progeny. Avian Dis.

[pmed-1000043-b022] Mitchell BW, Waltman WD (2003). Reducing airborne pathogens and dust in commercial hatching cabinets with an electrostatic space charge system. Avian Dis.

[pmed-1000043-b023] Mitchell BW, Buhr RJ, Berrang ME, Bailey JS, Cox NA (2002). Reducing airborne pathogens, dust and Salmonella transmission in experimental hatching cabinets using an electrostatic space charge system. Poult Sci.

[pmed-1000043-b024] Gast RK, Mitchell BW, Holt PS (1999). Application of negative air ionization for reducing experimental airborne transmission of Salmonella enteritidis to chicks. Poult Sci.

[pmed-1000043-b025] Nardell EA (1999). Air sampling for tuberculosis- homage to the lowly guinea pig. Chest.

[pmed-1000043-b026] Riley RL (1957). Aerial dissemination of pulmonary tuberculosis. Am Rev Tuber.

[pmed-1000043-b027] Riley RL, Mills CC, O'Grady F, Sultan LU, Wittstadt F (1962). Infectiousness of air from a tuberculosis ward. Ultraviolet irradiation of infected air: comparative infectiousness of different patients. Am Rev Resp Dis.

[pmed-1000043-b028] Noakes CJ, Sleigh PA, Escombe AR, Beggs CB (2006). Use of CFD analysis in modifying a TB ward in Lima, Peru. Indoor & Built Environment.

[pmed-1000043-b029] Moore DA, Evans CA, Gilman RH, Caviedes L, Coronel J (2006). Microscopic-observation drug-susceptibility assay for the diagnosis of TB. N Engl J Med.

[pmed-1000043-b030] Escombe AR, Oeser C, Gilman RH, Navincopa M, Ticona E (2007). The detection of airborne transmission of tuberculosis from HIV-infected patients, using an in vivo air sampling model. Clin Infect Dis.

[pmed-1000043-b031] Altman R, Altman D (2000). Statistical guidelines for contributors to medical journals. Statistics with confidence.

[pmed-1000043-b032] Riley RL, Knight M, Middlebrook G (1976). Ultraviolet susceptibility of BCG and virulent tubercle bacilli. Am Rev Respir Dis.

[pmed-1000043-b033] Ko G, First MW, Burge HA (2002). The characterization of upper-room ultraviolet germicidal irradiation in inactivating airborne microorganisms. Environ Health Perspect.

[pmed-1000043-b034] Rudnick SN, First MW (2007). Fundamental factors affecting upper-room ultraviolet germicidal irradiation - part II. Predicting effectiveness. J Occup Environ Hyg.

[pmed-1000043-b035] Ko G, Burge HA, Nardell EA, Thompson KM (2001). Estimation of tuberculosis risk and incidence under upper room ultraviolet germicidal irradiation in a waiting room in a hypothetical scenario. Risk Anal.

[pmed-1000043-b036] Nicas M, Miller SL (1999). A multi-zone model evaluation of the efficacy of upper-room air ultraviolet germicidal irradiation. Appl Occup Environ Hyg.

[pmed-1000043-b037] Stead WW (1989). Clearing the air: the theory and application of ultraviolet air disinfection. Am Rev Respir Dis.

[pmed-1000043-b038] Iseman MD (1992). A leap of faith. What can we do to curtail intrainstitutional transmission of tuberculosis. Ann Intern Med.

[pmed-1000043-b039] Macher JM, Alevantis LE, Chang YL, Liu K (1992). Effect of ultraviolet germicidal lamps on airborne microorganisms in an outpatient waiting room. Appl Occup Environ Hyg.

[pmed-1000043-b040] Brubacher J, Hoffman RS (1996). Hazards of ultraviolet lighting used for tuberculosis control. Chest.

[pmed-1000043-b041] Talbot EA, Jensen P, Moffat HJ, Wells CD (2002). Occupational risk from ultraviolet germicidal irradiation (UVGI) lamps. Int J Tuberc Lung Dis.

[pmed-1000043-b042] Nardell EA, Bucher SJ, Brickner PW, Wang C, Vincent RL (2008). Safety of upper room ultraviolet germicidal air disinfection for room occupants. Public Health Rep.

[pmed-1000043-b043] First MW, Weker RA, Yasui S, Nardell EA (2005). Monitoring human exposures to upper room germicidal ultraviolet irradiation. J Occup Environ Hygiene.

[pmed-1000043-b044] First MW (2007). Performance of UV germicidal irradiation lamps and luminaires in long-term service. Leukos.

[pmed-1000043-b045] Riley RL, Permutt S, Kaufman JE (1971). Convection, air mixing, and ultraviolet air disinfection in rooms. Arch Environ Health.

[pmed-1000043-b046] Riley RL, Permutt S (1971). Room air disinfection by ultraviolet irradiation of upper air. Air mixing and germicidal effectiveness. Arch Environ Health.

[pmed-1000043-b047] First M, Rudnick SN, Banahan KF, Vincent RL, Brickner PW (2007). Fundamental factors affecting upper-room ultraviolet germicidal irradiation - part I. Experimental. J Occup Environ Hyg.

[pmed-1000043-b048] Kerr KG, Beggs CB, Dean SG, Thornton J, Donnelly JK (2006). Air ionisation and colonisation/infection with methicillin-resistant Staphylococcus aureus and Acinetobacter species in an intensive care unit. Intensive Care Med.

[pmed-1000043-b049] Escombe AR, Moore DA, Gilman RH, Pan W, Navincopa M (2008). The infectiousness of tuberculosis patients co-infected with HIV. PLoS Med.

[pmed-1000043-b050] Balasubramanian V, Wiegeshaus EH, Smith DW (1992). Growth characteristics of recent sputum isolates of Mycobacterium tuberculosis in guinea pigs infected by the respiratory route. Infect Immun.

[pmed-1000043-b051] Nardell EA, Wallis RS (2006). Here today, gone tomorrow - the case for transient acute tuberculosis infection. Am J Resp Crit Care Med.

[pmed-1000043-b052] Basu S, Andrews JR, Poolman EM, Gandhi NR, Shah NS (2007). Prevention of nosocomial transmission of extensively drug-resistant tuberculosis in rural South African district hospitals: an epidemiological modelling study. Lancet.

