# Supplementary material for: Upper-Room Ultraviolet Light and Negative Air Ionization to Prevent Tuberculosis Transmission
Source: PLoS Med. 2009 Mar 17;6(3):e1000043. doi: 10.1371/journal.pmed.1000043 (PMC2656548; doi:10.1371/journal.pmed.1000043)
Supplement: Supplementary file 1 [file pmed.1000043.sd001.doc]

Text S1

**1. Wells-Riley Calculation Methodology**

The Wells-Riley equation for airborne infection is defined as[1]:

$C=S\left( 1-e^{-\text{qpt}Q} \right)$	(1)

where:	C = number of new cases

S = number of susceptibles exposed

q = infectious quanta produced per hour

p = pulmonary ventilation of susceptibles (m^3^/hour)

t = exposure time (hours)

Q = absolute ventilation of room (m^3^/hour)

The term “quantum” is used to describe the “infectious dose” for TB, defined as the number of infectious particles required to cause infection in (1 − *e*^−1^) of a susceptible population when each susceptible individual breathes, on the average, one quantum of infectious particles[15].

Rearranging this equation enables the calculation of infectious quanta production rates based on actual infection data and ventilation rate measurements.

$q=-\frac{Q}{\text{Ipt}}\text{ln}\left( 1-\frac{C}{S} \right)$	(2)

Equation (2) was initially applied to the results for each test period from the Control animal enclosure to find the effective quanta generation rate in that enclosure, *q_C_*. Duration of exposure was defined as the total number of days between PPD tests and the number of susceptibles was taken as the number of guinea pigs exposed during that time. Guinea pig pulmonary ventilation was assumed to be 0.23 m^3^/day[46] and the number of new cases was taken as the number of animals with TB infection (PPD positive conversions), or TB disease (autopsy or culture evidence of TB). Analysis was carried out using both the raw data and also data corrected for the dust related outbreak. The absolute ventilation, Q, was calculated from the mean of measurements of the total airflow leaving the animal house over the exposure period. Mean airflow (cubic feet per minute; cfm) into and out of the animal enclosures over the entire study was 212 (SD 24) and 221 (SD 26); 215 (SD 31) and 229 (SD 30); and 216 (SD 25) and 228 (SD 25) for the Control, UV and Ionizer enclosures respectively. Much of the airflow variance was due to seasonal fluctuation, which was the same for all three enclosures and is accounted for in the Wells-Riley analysis.

The effective quanta generation rate in the Control enclosure, q_c_, is related to the quanta generation rate on the ward, *q_w_* by the equation

$qc=\text{xq}_{w}$	(3)

where *x* is the proportion of the extracted ward air that passes through the Control animal enclosure. During the study, the airflow was split between the Control and Ionizer enclosures on ‘UV-off’ days. Assuming that the proportion of ward air to each enclosure is equal to the ratio of the measured airflow into the two enclosures enables the calculation of a mean value for *x* over each test period and hence *q_w_* in equation (3). It is also possible to calculate the effective quanta generation rate in the Ionizer enclosure, q_I_, which would be expected in the absence of the intervention, as

$q_{I}=(1-x)q_{w}$	(4)

With this expected quanta value, equation (1) can then be applied with the number of susceptibles and mean extract airflow over the test period for the Ionizer enclosure to find the expected number of new cases in this enclosure in the absence of the intervention. Furthermore, assuming that the mean quanta production on the ward over a test period is the same on ‘Control’ and ‘UV’ days, a similar calculation can be carried out for the UV enclosure using *q_w_* determined from the Control enclosure and *x* calculated from the ratio of the air entering the UV enclosure and that diverted through the by-pass duct to the exhaust chimneys.

The expected numbers of new cases in the absence of interventions were calculated for the UV and Ionizer enclosures for each period of the study. In each case the actual and expected % of animals infected were calculated for each study period as well as over the whole study to determine the overall % reduction in infections for the two enclosures due to the interventions alone. With correction for the dust related outbreak, the analysis based on TB infection yielded overall reductions of 70.4% and 59.7% for the UV lights and the negative ionizers respectively. Analysis based on the TB disease data resulted in calculated overall reductions of 53.5% and 50.9% for UV and ionizers respectively. Results for the uncorrected data were very similar in both cases.

As an example of the calculation process, measured and calculated data based on PPD positive results is presented below for the twelfth period of the study to show % reduction in infections due to the intervention without the influence of the airflow variation.

(a) Mean supply and extract air for the three animal enclosures and calculated ward air split, x, on ‘Control’ and ‘UV’ days

| **Test** | **Time** | **Air into enclosure (cfm)** | | | | **x** | | **Air extracted (cfm)** | | |
| --- | --- | --- | --- | --- | --- | --- | --- | --- | --- | --- |
|  | **(days)** | **Control** | **UV** | **Ionizers** | **bypass** | **Control** | **UV** | **Control** | **UV** | **Ionizers** |
| 12 | 30 | 210 | 208 | 218 | 241 | 0.5 | 0.5 | 223 | 222 | 246 |

(b) Calculated actual and expected quanta values and expected cases in the absence of intervention

| **PPD positives** | | | **Susceptibles** | | | **q_c_** | **qw** | **q_UV_** | **q_I_** | **Expected cases** | | |
| --- | --- | --- | --- | --- | --- | --- | --- | --- | --- | --- | --- | --- |
| **Control** | **UV** | **Ionizers** | **Control** | **UV** | **Ionizers** |  |  |  |  | **Control** | **UV** | **Ionizers** |
| 14 | 2 | 6 | 142 | 141 | 143 | 139 | 282 | 131 | 144 | 14 | 13 | 13 |

(c) Percentage of animals infected in each enclosure; actual values, expected values in the absence of intervention and reduction over test period due solely to the intervention

| **Actual (%)** | | | **Expected (%)** | | **Reduction (%)** | | |
| --- | --- | --- | --- | --- | --- | --- | --- |
| **Control** | **UV** | **Ionizers** | **UV** | **Ionizers** | **Control** | **UV** | **Ionizers** |
| 9.9 | 1.4 | 4.2 | 9.3 | 9.3 | 0 | 85 | 55 |

**2.** **Room air mixing and UV lamp effectiveness**

Ventilation air supplied to the isolation rooms followed a high supply –low extract approach, as shown in Figure A1. To evaluate the likely protection at a room level provided by upper-room UV lamps combined with mixing fans, zonal mixing models have been employed. These have been used in previous studies to evaluate ventilation influence on UV effectiveness[2]. Comparison with CFD modelling has shown that although they are not capable of representing high concentrations close to an infectious source, even a two zone model is a good representation of infectious aerosol concentrations and UV performance at a general room level [3].

Patient and bed

q

βQ

(1+β)Q

Q

Q

V_l_, C_l_

V_u_, C_u_

UV lamp

Q_f_

Fan

Figure A1: Schematic of ventilation regime, room air mixing and upper-room UV field.

The room is represented as two zones, where the upper-zone, V_u_ contains the UV lamp and the lower zone, V_l_ contains the patient who emits infectious quanta, q (quanta/hr) and any healthcare workers. The rooms considered in this study varied slightly in size so for the purposes of this analysis an average of 40 m^3^ is taken, with the upper UV zone 23% of this based on a lamp location of 0.7 m from the ceiling. The room is ventilated at a rate Q = 236m^3^/hr (5.9 AC/h) and air in each zone is assumed to be fully mixed while the mixing between zones is incomplete and is defined relative to the room ventilation rate by a factor β. The upper zone has an average UV irradiance E = 0.25 W/m^2^ which is determined from the average of 327 measurements taken at lamp level, ceiling level and at a point mid-way between the two. The infectious aerosol has a susceptibility to UV-C irradiation Z m^2^/J, which for TB is taken as 0.47 m^2^/J [4].

Applying the models described in [2] and [3] and evaluating the resulting upper and lower zone infectious quanta concentrations relative to the lower zone concentration in the absence of UV irradiation yields the results shown in Figure A2. This indicates how the relative concentration of each zone depends upon the mixing factor, β, within the space. As expected, as the mixing in the space increases, the upper and lower zone concentrations approach the same value, with the UV irradiated space at a lower relative concentration.

Figure A2: Influence of room mixing on infectious concentration.

In this case the mixing is due to a combination of the mixing fan and airflow within the space. The value of β is not easy to determine as it depends on the local airflow in the space, however it can be estimated from the prior CFD study of the ventilation air [5] and knowledge of the mixing fan. Considering the mixing fans alone, the volume flow rate through the fan is Q_f_ = 382 m^3^/hr, which is equivalent to the volume flow rate between the two zones of the room. Relative to the overall room ventilation rate this yields a fan mixing factor of β_f_ = 382/236 =1.62. The ventilation air mixing factor is estimated using the approach in [3] where the average vertical velocity at the interface between the upper and lower zones is found from the prior CFD study[5]. This yields a value of 0.038 m/s, which is equivalent to volume flow rate of 1838 m^3^/hr across the area of the interface. This value is the total flow over this interface, which from Figure A1 this is βQ+(1+β)Q = Q + 2βQ. Assuming 236 m^3^/hr is the downward component of the ventilation (Q), the remaining 1602 m^3^/hr is the inter-zonal mixing component (2βQ), half of which is directed upwards and half downwards. Following [3] and dividing by 2Q, this yields a ventilation mixing factor of β_v_ = 3.39. Hence the total mixing factor for the space is estimated as β_v_ + β_f_ = 5.01. Comparing this mixing factor with Figure A2 shows that the expected concentration in the lower zone is reduced by approximately 79% through the introduction of upper room UV lamps. This predicted reduction in concentration of infectious particles is consistent with the 70% reduction in guinea pig infections observed during the study. According to the model, with a mixing factor of 5 the room is 83% mixed. As a result the reduction in guinea pig infections is comparable to the likely protective effect for health-care staff and visitors in the room, except when they are in very close proximity to the patient.

**References:**

1.Riley, R.L. and E.A. Nardell, *Clearing the air. The theory and application of ultraviolet air disinfection.* Am Rev Respir Dis, 1989. **139**(5): p. 1286-94.

# 2.Noakes, C.J., Beggs, C.B. and Sleigh, P.A. (2004) Effect of Room Mixing and Ventilation Strategy on the Performance of Upper Room UV Germicidal Irradiation Systems, *Proceedings of* *ASHRAE IAQ 2004*, Tampa, Florida 15-17th March

3.Noakes, C.J., Beggs, C.B. and Sleigh, P.A. (2004) Modelling the Performance of Upper Room UV Germicidal Irradiation Devices in Ventilated Rooms: Comparison of Analytical and CFD Methods, *Indoor and Built Environment,* 13(6), 477-488

4.Riley, R.L., Knight M and Middlebrook G (1976). Ultraviolet susceptibility of BCG and virulent tubercle bacilli. *American Review of Respiratory Disease* 113: 413-418

5.Noakes, C.J., Sleigh, A., Escombe, R., Beggs, C.B. (2006) Use of CFD Analysis in Modifying a TB Ward in Lima, Peru. *Indoor and Built Environment*, 15(1), 41-47.
